# Methylmalonic aciduria as a biochemical marker for mitochondrial DNA depletion syndrome in patients with developmental delay and movement disorders: a case series

**DOI:** 10.3389/fneur.2023.1265115

**Published:** 2023-11-24

**Authors:** Montaha Almudhry, Arushi Gahlot Saini, Mohammed A. Al-Omari, Yashu Sharma, Maryam Nabavi Nouri, C. Anthony Rupar, Chitra Prasad, Andrea C. Yu, Savita Verma Attri, Asuri Narayan Prasad

**Affiliations:** ^1^London Health Sciences Centre, London, ON, Canada; ^2^Department of Neuroscience, King Fahad Specialist Hospital, Dammam, Saudi Arabia; ^3^Department of Pediatrics, Postgraduate Institute of Medical Education and Research, Chandigarh, India; ^4^Department of Pediatrics, College of Medicine, Imam Abdulrahman Bin Faisal University, Dammam, Saudi Arabia; ^5^King Fahad Hospital of the University, Al-Khobar, Saudi Arabia; ^6^Department of Pediatrics, University of Western Ontario, London, ON, Canada; ^7^Department of Clinical Neurological Science, Western University, London, ON, Canada; ^8^Department of Biochemistry, University of Western Ontario, London, ON, Canada; ^9^Department of Pediatrics, Medical Genetics Program of Southwestern Ontario, London, ON, Canada; ^10^Department of Pediatrics, Children’s Hospital of Eastern Ontario, Ottawa, ON, Canada

**Keywords:** methylmalonic acid, mitochondrial DNA depletion syndrome, *SUCLG1*, *SUCLA2*, mitochondrial disorder, dystonia, movement disorder

## Abstract

**Background:**

Mitochondrial DNA (mtDNA) depletion syndromes (MDDS) are genetically and clinically variable disorders resulting from a reduction in mtDNA content in the cells, tissues, and organ systems, leading to symptoms related to energy deficits. Deficiency of the mitochondrial succinyl-CoA ligase/synthetase enzyme secondary to pathogenic variations in the *SUCLG1* and *SUCLA2* genes is a subtype of MDDS that presents with neurological manifestations and a specific biochemical profile.

**Methods:**

This cross-sectional series describes five patients with MDDS secondary to pathogenic variations in the *SUCLG1* and *SUCLA2* genes from two tertiary care centers in Canada and India. Clinical data concerning the course, investigations, and outcome were gathered through chart reviews.

**Results:**

All subjects presented in early infancy with neurological manifestations, including movement disorder, psychomotor regression, developmental delay, hearing loss, behavioral issues, or a combination thereof. Elevated methylmalonic acid metabolites, an abnormal acylcarnitine profile, and lactic acidemia were noted in the biochemical profile of each patient (*n* = 5/5, 100%). Molecular genetic testing disclosed the presence of pathogenic homozygous mutations in four subjects and compound heterozygosity in one subject.

**Conclusion:**

MDDS associated with *SUCLG1* and *SUCLA2* genes can be detected biochemically by the presence of methylmalonic aciduria besides the elevation of lactate, C3, C4DC, and C5-OH acylcarnitine. Conducting metabolic workups including MMA and acylcarnitine profiles in patients with heterogeneity of clinical symptoms associated with the presence of this biochemical marker may potentially reduce the time to diagnosis and management.

## Introduction

Mitochondrial DNA depletion syndromes (MDDS) are a group of severe neurometabolic diseases characterized by a reduction in the amount of mitochondrial DNA (mtDNA) in cells. These disorders are caused by mutations in the genes involved in mtDNA replication, maintenance, and repair and may have autosomal recessive, maternal, or X-linked inheritance patterns (1). In some situations, mutations in genes involved in mtDNA replication, such as *POLG*, *TK2*, and *DGUOK*, lead to impaired mtDNA synthesis and a consequent reduction in the amount of mtDNA in cells. In others, mutations in genes involved in mtDNA maintenance and repair, such as *TWINKLE* and *OPA1*, can lead to mtDNA depletion by impairing the stability and integrity of mtDNA.

*SUCLG1*-related MDDS 9 (encephalomyopathic type with methylmalonic aciduria, OMIM #245400) and *SUCLA2*-related MDDS 5 (encephalomyopathic type with or without methylmalonic aciduria, OMIM #612073) are rare subtypes of MDDS caused by biallelic pathogenic variations in the *SUCLG1* and *SUCLA2* genes. These genes, respectively, encode for α and β subunits of the mitochondrial matrix enzyme succinyl-CoA synthetase (also known as succinate ligase). The latter enzyme catalyzes the reversible conversion of succinyl-CoA to succinate and coenzyme A and participates in the conversion of ADP to ATP and GDP to GTP. The β-subunit is also encoded by the *SUCLG2* gene. While *SUCLG1* is expressed ubiquitously across all tissues, *SUCLA2* is predominantly expressed in the brain, heart, and muscle, and *SUCLG2* is restricted primarily to the liver and kidney. The lack of either subunit of the succinyl-CoA synthetase leads to the accumulation of succinyl-CoA, which is metabolized to methylmalonic acid (MMA) and accumulates in the body fluids (blood and urine), thus providing a biochemical marker that can lead to the diagnosis.

In the present case series, we describe patients with MDDS secondary to pathogenic variants in the *SUCLG1* and *SUCLA2* genes who had mitochondrial encephalopathy, lactic acidosis, and elevated MMA in urine. These cases underscore the significance of MMA detection in urine organic acid analysis and can serve as a useful biochemical marker in cases with varied neurological presentations (dystonia, developmental delay, other movement disorders, and hearing loss).

## Methods

This cross-sectional series describes five children with MDDS from two tertiary care pediatric centers in Canada and India. Clinical presentation, family history, biochemical profile, radiological features, genetic, and treatment data were extracted from electronic and paper case records. Neuroimaging features and outcomes were assessed. The data were compiled on an Excel spreadsheet. The study meets the requirements of the Human Research Ethics Board (HREB) at Western University, London, Canada, and the corresponding committee at the Post Graduate Institute of Medical Education and Research, Chandigarh, India.

## Results

A total of five patients were included in this series. All of them presented within the first year of life (median age of presentation: 6 months, range: 4–6 months). The majority were boys (male:female ratio: 4:1). The individual case details are described below, and the comparative findings are reported in Table 1. All individuals presented with developmental problems and a hyperkinetic movement disorder (5/5, 100%). Three of the five children were symptomatic with hearing loss, while only one manifested with optic atrophy and one with ophthalmoplegia. Hydrocephalus was noted in two of our patients; however, none of our subjects experienced epileptic seizures. Urinary MMA was elevated in all the patients (*n* = 5, 100%). An abnormal acylcarnitine profile and lactic acidemia were also noted in each individual in the cohort. The most frequently detected abnormalities were elevated C3, C5-OH, and C4DC-acylcarnitine. In one child (20%), neuroimaging was reported as normal, while in the other four (80%), signal abnormalities were identified in the basal ganglia and white matter. The outcomes in this series were variable but included persisting extrapyramidal features in the affected children. When comparing the outcomes of MDDS associated with *SUCLA2* and MDDS associated with *SUCLG1*, the former was associated with a relatively milder degree of neurologic impairment.

**Table 1:** Comparison of neurological manifestations in the current study (*n* = 5).

	Case 1	Case 2	Case 3	Case 4	Case 5
Age of onset	6 months	4 months	5 months	6 months	6 months
Gender	Girl	Boy	Boy	Boy	Boy
Clinical features	Language delay, followed by developmental regression, dyskinetic movement disorder, and sensorineural hearing loss	Global developmental delay, central hypotonia, choreoathetoid movements, and ophthalmoplegia	Global developmental delay and generalized dystonia	Developmental delay followed by regression, central hypotonia, optic atrophy, and hearing loss	Normal development followed by developmental arrest and generalized dystonia
Episodic encephalopathy	None	None	None	None	None
Seizures	None	None	None	None	None
Hydrocephalus	None	None	None	Present	Present
Consanguinity	None	None	None	None	None
Lactic acidosis	Present	Present	Present	Present	Present
Acylcarnitine profile	Elevated C3 and C4DC-Acylcarnitine	Elevated C3 and C4DC-Acylcarnitine	Elevated C3, C5-OH, and C4DC-Acylcarnitine	Elevated levels of methyl malonyl carnitine and hydroxy isovaleryl carnitine	Elevated C5OH and C4DC-Acylcarnitine
Urine MMA	Present	Present	Present	Present	Present
Age at MRI	9 years	14 months	1 year	1 year	2 years
Brain MRI	Unremarkable	Bilateral caudate, putamen, and globi pallidi hyperintensity	Bilateral caudate and lentiform nuclei hyperintensity Periventricular white matter hyperintensity	Bilateral caudate, lentiform nuclei hyperintensity, and atrophy Hydrocephalus	Bilateral globus pallidi, caudate and putamen hyperintensity, and atrophy Hydrocephalus Diffuse cerebral atrophy Periventricular and subcortical white matter hyperintensity
Genetic mutation	Homozygous missense mutation in *SUCLA2* gene. c.985A > G, p.(Met329Val)	Compound heterozygous mutation in *SUCLG1* gene. (i) c.201G > A, p. (Gln67), (ii) c.825 + 4A > T	Homozygous missense mutation in *SUCLG1* gene c.358G > C (p.Val120Leu)	Homozygous missense mutation in *SUCLG1* gene c.358G > C (p.Val120Leu)	Homozygous missense mutation in *SUCLG1* c.358G > C (p.Val120Leu)
Therapeutic interventions	Mitochondrial cocktail	Supportive symptomatic management	Oral and injectable B_12_, carnitine, clonazepam, and trihexyphenidyl	Mitochondrial cocktail, B_12_ injections, trihexyphenidyl, baclofen, and bilateral pallidotomy	Trihexyphenidyl, baclofen, carnitine, coenzyme Q, and oral B_12_
Follow-up duration	14 years	4 years	2 years	2 years	3 years
Outcome	Slow steady gain of milestones, intermittent dyskinesia, no regression, and ambulatory	Global developmental delay with speech regression after 1 year of age but steady gains in other domains, recurrent vomiting	Severe generalized dystonia, spastic quadriparesis, severe developmental delay, and non-ambulatory	Severe generalized dystonia, severe developmental delay, and non-ambulatory	Intermittent dystonia, lower limb spasticity, slow gain of milestones, non-ambulatory, and difficulty sleeping

### Case 1

A 13-year-old girl presented for evaluation of developmental delay and intermittent fidgety movements. She was noted to have a language-predominant delay and presented with an acute loss of motor skills, generalized hypotonia, and bulbar dysfunction without any apparent prodrome or febrile illness at 6 months of age. She showed a gradual recovery from this episode. At 18 months of age, she was diagnosed with bilateral mild-to-moderate sensorineural hearing loss, requiring hearing aids. Subsequently, her language and communication skills improved. By 2 years of age, she developed choreoathetoid movements in bilateral distal upper limbs and an abnormal gait and was diagnosed with athetoid cerebral palsy. She remained static in her illness until the age of 13, with intermittent fluctuations during sickness or fatigue. The parents additionally reported prolonged recovery time following anesthesia, where she would remain motionless even though she was awake. She was born at term following an uneventful pregnancy and an uncomplicated delivery. Family history showed a significant prevalence of learning disabilities and attention deficit hyperkinetic syndrome among the paternal first-degree relatives, but no consanguinity, deafness, developmental regression, or movement disorders were noted.

An initial examination at 4 years of age showed a weight of 12.25 kg (15th percentile), a height of 94.5 cm (below the 10th percentile), and a head circumference of 49 cm (25th percentile). Her facial appearance was long, and a thin upper lip was noted. Neurologic examination showed hypotonia with relatively preserved muscle power, choreoathetosis in the distal upper extremities, brisk deep tendon reflexes, ankle clonus, cerebellar ataxia, and poor coordination. The remainder of the systemic examination was not contributory.

Investigations showed a normal magnetic resonance imaging (MRI) scan of the brain at 9 years of age and a normal newborn screening. Her biochemical profile at the age of 8 years showed elevated lactic acid (5.1 mmol/L, normal 0.5–2.2) and the presence of MMA on urine organic acid analysis. The acylcarnitine profile detected a markedly increased level of C3 (2.90 μmol/L, range < 0.65 μmol/L) and a small increase of C4DC acylcarnitine (0.39 μmol/L, range < 0.10 μmol/L). Based on the biochemical profile, a mitochondrial nuclear gene panel test was ordered at the age of 8 years. The test results disclosed a homozygous, missense variant c.985A > G, p. (Met329Val) in the *SUCLA2* gene, confirming the diagnosis of succinate-CoA ligase deficiency-related MDDS. The variant was categorized as pathogenic based on *in silico* analysis. A mitochondrial cocktail was initiated, and the child received coenzyme Q10 240 mg daily, carnitine 500 mg TID, vitamin B1 100 mg daily, levocarnitine 500 mg BID, B12 500 μg daily, and B6 100 mg daily. At 13 years of age, she continues to gain milestones slowly with independent ambulation but otherwise remains symptomatic with intermittent dystonic posturing, dyskinesias, and mood abnormalities during intercurrent illnesses.

### Case 2

A 4-year-old boy presented with global developmental delay. At the age of 4 months, his parents noted that he was floppy with poor head control. At 10 months of age, he was assessed to have global developmental delay (with a developmental age of 4 months) with central hypotonia. There was no history of abnormal movements, vision or hearing abnormalities, dystonic posturing, or seizures. He was born full-term to non-consanguineous parents, following an uneventful perinatal course. Family history was not contributory. Examination at 10 months of age showed a normal head circumference of 45 cm (36th percentile), weight of 8.2 kg (4th percentile), and length of 74.5 cm (11th percentile). He had bilateral epicanthal folds, an upturned nose, a short columella, a long philtrum with a thin upper lip, and slightly low-set ears. The neurological exam was significant for the limitation of upward gaze (60° upward gaze) axial hypotonia with increased tone in the upper extremities. Reflexes were depressed in the lower extremities (+1/4 in the knees and ankles). The remainder of the systemic examination was unremarkable.

Chromosomal microarray, thyroid function, blood gases, ammonia, and CK levels were within normal limits. A urine organic acid assay detected the presence of MMA. Serum MMA was 2.56 μmol/L (0.1–0.4). The acylcarnitine profile showed elevated methylmalonyl and succinyl C4DC at 0.23 μmol/L (normally less than 0.13 μmol/L) and elevated propionyl C3 at 1.60 μmol/L (normally less than 1.08 μmol/L). An MRI of the brain done at 14 months of age revealed an abnormal signal with T2/FLAIR signal hyperintensity in the bilateral caudate nuclei, putamen, and globi pallidi. MR spectroscopy was unremarkable.

Analysis of the mitochondrial genome and nuclear genes showed three variants of uncertain significance. Two of them were in the *SUCLG1* gene: c.201G > A, p. (Gln67=) and c.825 + 4A > T, and m.9759\u00B0C > T. p. (Pro185Ser), and a third variant in the *MT-CO3* gene. *In silico* analysis using Net Gene2 and Human Splicing Site Finder predicted that these compound heterozygous variants affected splicing and were classified as pathogenic (ACMG category 3). The third variant presented near homoplasmy. It is not described as disease-causing in the MitoMap database and is likely benign in the ClinVar database. Overall, the clinical, biochemical, and molecular genetic data supported the diagnosis of succinate-CoA ligase deficiency (*SUCLG1*-related MDDS). He continues to remain developmentally delayed with speech regression but continues to gain steadily in other domains (gross motor, fine motor, and adaptive). Recurrent hospitalizations related to symptomatic episodes of emesis, gait unsteadiness, and lactic acidemia with subsequent recovery have been a feature of his progress to date.

### Case 3

A 12-month-old boy presented with global developmental delay and progressive, generalized dystonia. He was first noticed to have a global delay by the age of 5 months, when he had a social smile and could only hold his head. Following an episode of lower respiratory tract infection at the same age, he developed generalized limb weakness and impaired movements. He gradually recovered and developed progressive generalized dystonia in the following months. He was the third child of a non-consanguineous union after an uneventful pregnancy and was born with a normal birth weight. Family history was not contributory. One sibling passed away in the neonatal period (day 3 of life) due to congenital heart disease and esophageal atresia. Examination showed spastic quadriparesis, brisk deep tendon reflexes, axial hypotonia with no head holding, generalized dystonia, excessive drooling, poor visual fixation and following impaired hearing, and normal head circumference (44 cm at 1 year of age). He had large ears with a prominent long philtrum, Mongolian spots over the back, and overriding of the second toe in the feet. Investigations disclosed a low mean corpuscular volume (69.5 fl), the absence of ketones, elevated lactate (3.3 mmol/L), normal ammonia, and a low pH (7.3). Tandem mass spectroscopy (TMS) showed moderately elevated propionyl carnitine (C3) (5.6 μM, range 0.18–3.12 μM), 3-hydroxyisovaleryl-2-methyl-3-hydroxybutyrylcarnitine (1.1 μM, C_5_OH/C_4_DC), C3/C2 ratio 0.37, and normal amino-acid levels, including those for methionine (12.9 μM, range 5–37 μM). Urine analysis by gas chromatography (GCMS) showed a mild elevation of MMA (2.37-fold). An MRI brain at 1 year of age showed bilateral, symmetrical T2/FLAIR hyperintensities in caudate and lentiform nuclei in the absence of contrast enhancement or diffusion restriction. There was no lactate peak identified on MR spectroscopy. A few T2/FLAIR hyperintensities were also seen in bilateral periventricular areas. Genetic analysis showed a pathogenic, homozygous, missense variation of c.358G > C (p.Val120Leu) in exon 4 on chromosome 2 in the *SUCLG1* gene. The identified variant was found in the CoA binding domain of the *SUCLG1* protein and resulted in the amino acid substitution of leucine for valine at codon 120 (p.Val120Leu; ENSTOOO0393868). Both biological parents were identified as carriers. The *in silico* analysis of the variant were possibly damaging by PolyPhen-2 (HumDiv and HumVar) and damaging by likelihood ratio test (LRT), Sorting Intolerant From Tolerant (SIFT), and Mutation Taster2. He was treated with B_12_ injections (1 mg/day), oral methylcobalamin (500 μg/day), carnitine 250 mg BID, clonazepam 0.25 mg TDS, and trihexyphenidyl 2 mg QID for the management of dystonia. In follow-up, he has severe generalized dystonia, failure to thrive, spastic quadriparesis, and severe developmental delay.

### Case 4

A 12-month-old boy presented with global developmental delay (motor > cognition) noted in early infancy, later followed by neurodevelopmental regression. He gradually lost auditory responsiveness and visual attention. There was no history of seizures. Birth and perinatal period were uneventful. He was the second child of a non-consanguineous marriage, and there was no history of similar illness in the family. On examination, he was irritable with no visual fixation or auditory attention. Head circumference was normal (43.5 cm). There was truncal hypotonia, an absence of neck holding, brisk deep tendon reflexes, a bilateral striatal toe, and an absence of organomegaly. Fundus examination showed optic atrophy.

**Figure 1:**
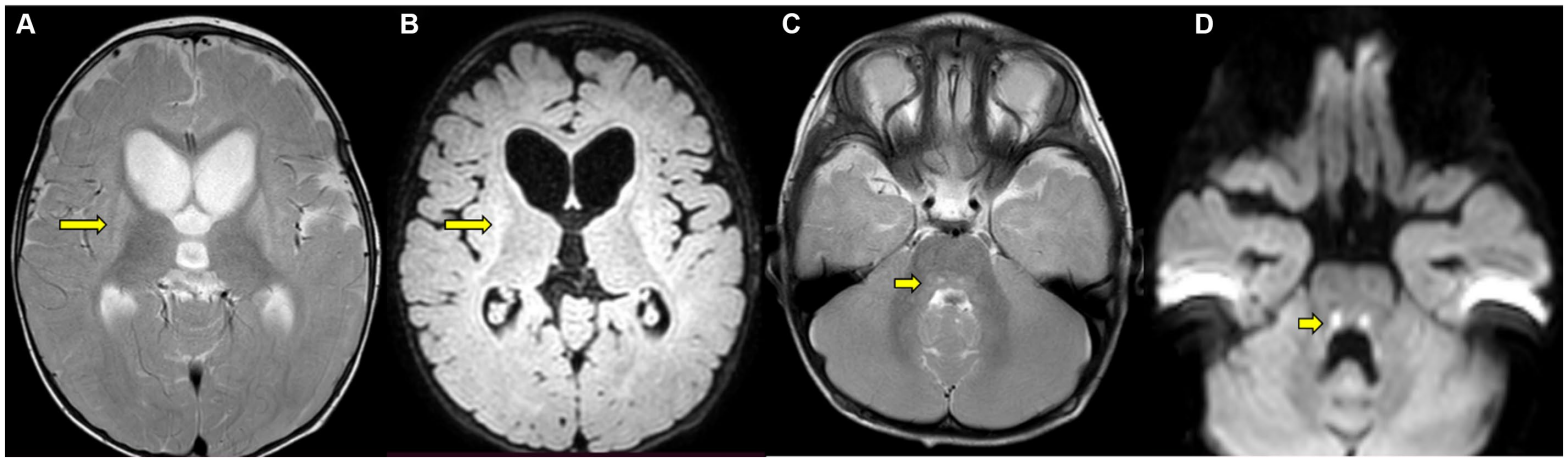
**(A–D)** MRI brain at 1 year of age in case 4. **(A)** Axial T2 and **(B)** FLAIR sections at the level of the basal ganglia show atrophy and hyperintensity in the head of the caudate nucleus and lentiform nuclei bilaterally (arrow) with hydrocephalus **(C)** T2-axial section at the level of the pons shows hyperintensity in central tegmental tract with **(D)** diffusion restriction (arrow).

Biochemical investigations showed elevated lactate (2.4 mmol/L). TMS showed elevated levels of methyl malonyl carnitine (1.16 μmol/L, range < 1) and hydroxy isovaleryl carnitine. All amino acid levels, including those for methionine (8.18 μmol/L, range 4.63–48), were normal. GCMS showed elevated MMA in urine. The MRI brain at 1 year of age showed atrophy and T2/FLAIR hyperintensity in the head of the caudate nucleus and lentiform nuclei bilaterally, with the absence of diffusion restriction and the presence of hydrocephalus (Figures 1A–C). T2 hyperintensity with diffusion restriction in the central tegmental tract was additionally noted (Figure 1D).

Whole exome sequencing identified a pathogenic, homozygous, missense mutation, c.358G > C (p.Val120Leu), in exon 4, chromosome 2 of the *SUCLG1* gene, resulting in an amino acid substitution of leucine for valine at codon 120 (p.Val120Leu). The *in silico* analysis of the variant were probably damaging by PolyPhen-2 (HumDiv) and damaging by SIFT, LRT, and MutationTaster2. Both parents were identified as carriers. He was started on coenzyme Q_10_ 300 mg daily, carnitine 500 mg daily, thiamine 100 mg BID, riboflavin 100 mg daily, vitamin C 500 mg daily, B_12_ injection 1 mg daily, trihexyphenidyl 2 mg QID, and baclofen 5 mg TDS. He underwent bilateral pallidotomy for worsening dystonia with limited benefit. At follow-up, he continues to have persistent generalized dystonia and remains non-ambulatory with excessive teeth grinding and feeding problems. His visual fixation and auditory attention have improved, but there has been no gain in motor milestones.

### Case 5

**Figure 2:**
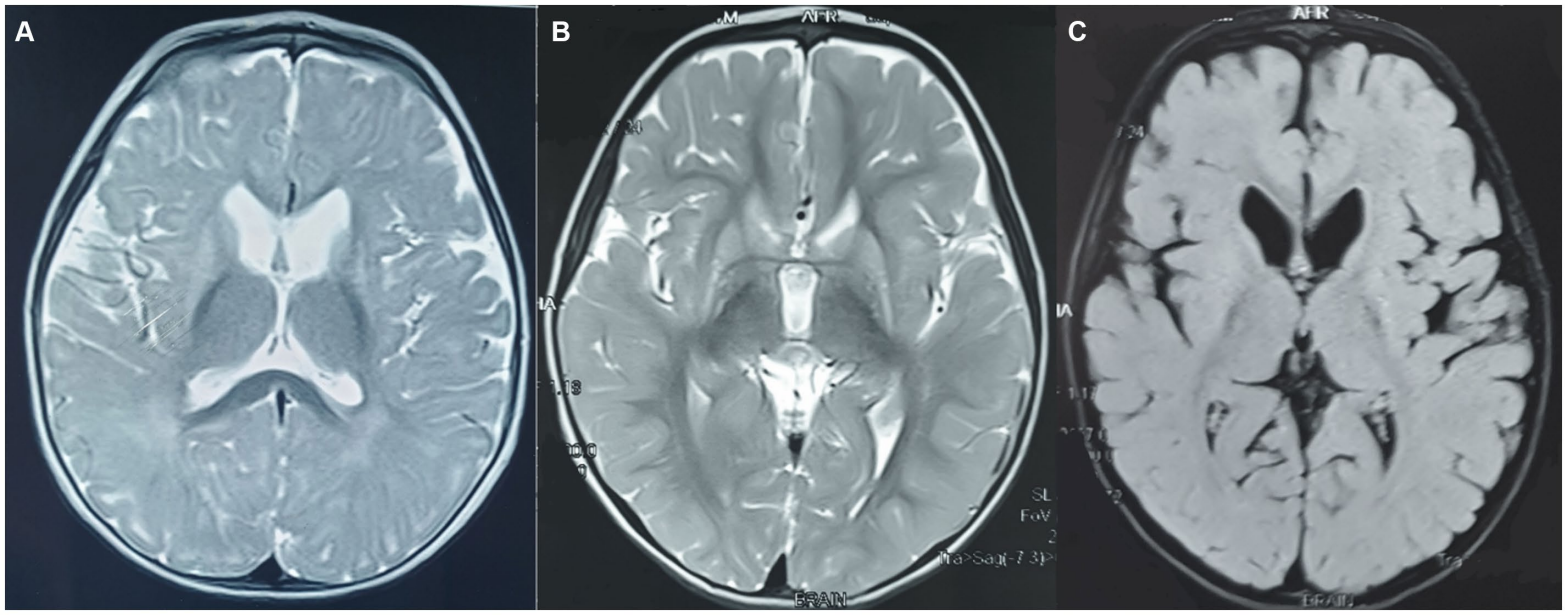
**(A–C)** MRI brain at 2 years of age in case 5. **(A)** T2-axial view at the level of the basal ganglia and **(B)** third ventricle show atrophy and hyperintensity in the head of the caudate nucleus and lentiform nuclei bilaterally with hydrocephalus **(C)** FLAIR axial sections show similar changes.

A 3-year-old boy presented with developmental delays and generalized dystonia. He was apparently well until 6 months of age. Subsequently, following an episode of excessive irritability lasting for a few days without fever, he developed developmental arrest and persistent irritability. Gradually, intermittent dystonia was noticeable in both upper limbs, which then became progressive. After a gap of several months, he began to regain milestones gradually. He had no history of seizures, altered sensorium, skin rashes, or vision impairment. His perinatal period was uneventful, with a normal birth weight. He was the second-child of a non-consanguineous marriage. No history of similar illnesses in the family was observed. Examination at 1 year of age showed axial hypotonia, persistent cortical thumb sign, lower limb spasticity, brisk deep tendon reflexes, normal hearing, normal head circumference (43 cm), and normal fundi bilaterally. Biochemical investigations disclosed the presence of lactic acidosis, normal C3 (3.3 μM, range 0.08–5 μM), C3/C2 0.18, and elevated levels of 3-hydroxyisovaleryl-2-methyl-3-hydroxybutyrylcarnitine (1.83 μM, range 0–0.65 μM, C_5_OH/C_4_DC) on TMS. The amino acid levels, including those for methionine (13.7 μM, range 5–75 μM), were normal. Urine organic acid analysis showed elevated MMA. MRI imaging at 2 years of age showed mild diffuse cerebral atrophy and hyperintensities in bilateral globus pallidi, caudate, and putamen on T2/FLAIR sequences without diffusion restriction, dilated frontal horns of lateral ventricles, focal bilateral symmetrical hyperintensities in the periventricular and subcortical white matter of temporal and frontal lobes, and normal MR spectroscopy (Figures 2A–C). Whole exome sequencing confirmed the presence of homozygous missense variant c.358G > C (p.Val120Leu) in exon 4 on chromosome 2 in the *SUCLG1* gene. Both parents were identified as carriers. The *in silico* analysis of the variant are deleterious by PolyPhen-2 (HumDiv and HumVar) and damaging by LRT, SIFT, and the mutation Taster2. He was initiated on trihexyphenidyl 2.5 mg QID, baclofen 5 mg TDS, carnitine 500 mg daily, coenzyme Q_10_ 300 mg daily, and oral vitamin B_12_ (500 μg daily) supplementation. At follow-up, he is non-ambulatory, has difficulty sleeping, and has constipation. He has intermittent, generalized dystonia and lower limb spasticity.

## Discussion

**Figure 3:**
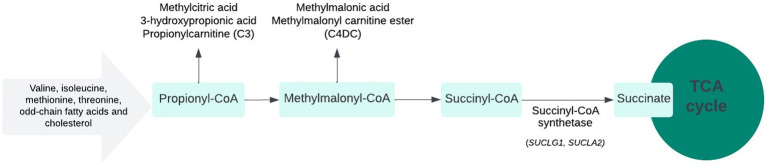
*SUCLA2* and *SUCLG1*-related metabolic pathway.

The present series highlights the significance of elevated urinary MMA as a marker of underlying MDDS in the context of developmental delay, regression, and movement disorder. Since the initial clinical presentation can be non-specific, the identification of such biochemical markers can aid early diagnosis and guide appropriate interventions and testing. MMA excretion in the urine can be due to or related to different genetic biochemical defects. It can be seen in association with a defect in branched-chain amino acid catabolism, a defect in the gene coding for methylmalonic mutase (several 100-fold elevations), or a defect in the synthesis of adenosylcobalamin, a coenzyme cofactor. The presence of this biochemical marker can indicate a differential diagnosis that includes propionic acidemia, MMA, inherited cobalamin defects and transport, multiple carboxylase deficiency, and B_12_ deficiency (2). Additionally, this finding could also be related to a deficiency in succinyl-CoA ligase, an enzyme in the mitochondrial matrix that results in MDDS, which otherwise results in mildly increased MMA excretion in the urine (3). Its presence in the blood is indicated by an elevated propionyl acylcarnitine level (C3). When C3 is only moderately elevated, the presence of secondary markers (C4DC and C3/C2 ratio > 0.2), low levels of methionine in the blood, and mild elevation of MMA in the urine suggest the diagnosis of MDDS (Figure 3) (4).

Classic patients with elevated MMA secondary to a catabolic defect in the branch-chain amino acid pathway present in the neonatal period/infancy with metabolic decompensation and encephalopathy, positive newborn screening (when available), and markedly elevated MMA in the urine make the detection of these causes relatively easier. On the other hand, in MDDS, due to *SUCLG1* and *SUCLA2* gene variations, patients present with variable constellations of neurologic symptoms and less marked metabolic decompensations. The elevation of MMA is only mild, and NBS could be normal, requiring a higher index of suspension and often repeated analysis of the acylcarnitine and urine organic acid profiles to detect biochemical abnormalities. In a Chinese study of 310 isolated methylmalonic acidemia patients, approximately 3.2% of patients were found to harbor a mutation in *SUCLG1*, while only 0.9% of them were secondary to the *SUCLA2* mutation (5). Furthermore, in a series of 25 patients with the *SUCLG1* mutation, 21 cases (84%) had mildly elevated MMA levels (6). This finding was also replicated by Carrozzo et al. in a larger cohort of 71 patients with succinate-CoA ligase deficiency (7). Thus, the presence of MMA in succinyl–coenzyme A synthase deficiency represents a highly sensitive screening test for MDDS related to *SUCLA2* and *SULG1* gene mutations in patients presenting with non-specific and variable neurological presentation and lactic acidosis.

As noted in previous published reports (7, 8), cases in this series presented with an early infantile-onset presentation, with variable neurologic manifestations including involuntary movement disorder, psychomotor regression, developmental delay, and hypotonia. These were followed by a slower developmental trajectory or severe psychomotor delay with significant comorbidities that include dystonia, behavioral issues, hearing loss, and sleep abnormalities. Epilepsy has also been described, more commonly in association with *SUCLA2* than *SUCLG1* gene mutations; however, none of the patients in this series developed epileptic seizures or evident encephalopathy. On the other hand, early-onset hydrocephalus was seen in two of our patients and is of significance, a finding that has not been previously reported. Hydrocephalus has been reported as a serious complication of MMA combined with homocysteinemia (9). The underlying pathophysiology is obscure. The proposed pathophysiological mechanisms include reduced arterial compliance and endothelial injury by the metabolites (such as homocysteine) that propagate the pulse pressure, which is transmitted to the cerebrospinal fluid (10). This causes ventricular dilation and higher intraventricular pulse pressure, thereby causing hydrocephalus. The latter may improve with metabolic intervention. It is worthwhile to monitor the evolution of early-onset hydrocephalus in MDDS patients. Although both types of *SUCLG1*- and *SUCLA2*-related MDS are present in infancy, the clinical course of *SUCLG1*-related MDS appears to be of greater severity and is progressive in nature.

In this series, we identified three novel variants in the *SUCLG1* gene (c.358G > C, c.201G > A, and c.825 + 4A > T). The homozygous missense variant, c.358G > C, was common in three patients (cases 4, 5, and 6) and caused the substitution of leucine for valine at codon 120 (p.Val120Leu), which led to a change in protein structure. These variants were deleterious or pathogenic in different prediction models like PolyPhen-2 (HumDiv and HumVar), LRT, SIFT, and Mutation Taster2 and have not been previously reported in exome/genome databases. The variant c.985A > G found in the *SUCLA2* gene (c.985A > G) has been reported earlier. In a study from Portugal, it was found that fibroblasts of a child with the c.985A > G variant demonstrated a reduced amount of *SUCLA2* protein production that leads to impaired mitochondrial ATP production and enhanced reactive oxygen species, thus impacting the disease prognosis (11). The genetic information, along with clinical and metabolic data, supports the diagnosis of succinate-CoA ligase deficiency-related MDDS.

There are no specific treatments available for this group of conditions. The following evaluations are suggested, with frequency varying according to the severity of the condition: routine developmental and neurologic assessment; monitoring for hydrocephalus; periodic nutritional and growth assessment; periodic hearing and ophthalmologic assessments; and regular examination of the back and joints for kyphoscoliosis and joint contractures (12). A mitochondrial cocktail using different antioxidants has been tried in these children anecdotally, despite the lack of systematic studies or evidence-based data regarding efficacy. No improvements could be documented in these patients.

## Conclusion

The diagnosis of MDDS is challenging, especially in young infants and children, due to the non-specific neurological presentation. Clinically, children with these disorders commonly present in infancy with non-specific features such as unexplained developmental delay, movement disorder, psychomotor regression, and hypotonia. These may be punctuated with acute deteriorations or may progress slowly as significantly delayed development and significant comorbidities, including severe dystonia, behavioral issues, hearing loss, and sleep abnormalities. The radiological presentation of bilateral basal ganglia involvement is an important clue toward an underlying neurometabolic disorder. Lactic acidosis, urinary excretion of MMA, and elevation of propionylcarnitine (C3) and C4DC acylcarnitine are important biochemical clues to these disorders. Awareness of doing a metabolic workup in otherwise non-specific neurologic symptomatology, particularly urine organic acids, and detection of urine MMA as a marker, would be helpful for pediatric neurologists. This could potentially shorten the time to diagnose patients. Early-onset hydrocephalus should be added to the clinical findings associated with *SUCLG1*-related MDDS. Appropriate genetic counseling for these autosomal recessive inherited diseases will be important for families.

## Data availability statement

The original contributions presented in the study are included in the article/supplementary material, further inquiries can be directed to the corresponding author.

## Ethics statement

The requirement of ethical approval was waived by the Human Research Ethics Board (HREB) at Western University, London, Canada for the studies involving humans because the number of participants is less than 5, and therefore we were exempt by the Human Research Ethics Board (HREB) at Western University, London, Canada. However, local approval was obtained from the Ethical Committee at the Post Graduate Institute of Medical Education and Research, Chandigarh, India. The studies were conducted in accordance with the local legislation and institutional requirements. Written informed consent for participation in this study was provided by the participants’ legal guardians/next of kin. Written informed consent was obtained from the minor(s)’ legal guardian/next of kin for the publication of any potentially identifiable images or data included in this article.

## Author contributions

MA: Writing – original draft, Writing – review & editing. AS: Formal analysis, Writing – original draft, Writing – review & editing. MA-O: Writing – original draft. YS: Writing – original draft, Writing – review & editing. MN: Supervision, Writing – review & editing. CR: Supervision, Writing – review & editing. CP: Supervision, Writing – review & editing. AY: Supervision, Writing – review & editing. SA: Supervision, Writing – review & editing. AP: Methodology, Project administration, Supervision, Writing – review & editing.
